# Optimization of Alfalfa-Based Mixed Cropping with Winter Wheat and Ryegrass in Terms of Forage Yield and Quality Traits

**DOI:** 10.3390/plants11131752

**Published:** 2022-06-30

**Authors:** Poe Thinzar Bo, Yongli Dong, Ruifang Zhang, Maw Ni Soe Htet, Jiangbo Hai

**Affiliations:** 1 State Key Laboratory of Crop Cultivation and Farming System in Northwestern Loess Plateau, College of Agronomy, Northwest A&F University, Yangling, Xianyang 712100, China; poethinzarbo@nwafu.edu.cn (P.T.B.); 2008114433@nwafu.edu.cn (Y.D.); 2019050002@nwafu.edu.cn (R.Z.); mawni2018071063@nwafu.edu.cn (M.N.S.H.); 2 State Key Laboratory of Crop Stress Biology for Arid Areas, College of Agronomy, Northwest A&F University, Yangling, Xianyang 712100, China; 3 Rice Bio-Park Research Section, Post-Harvest Technology and Food Science Research Division, Department of Agricultural Research, Yezin, Zayarthiri Township, Nay Pyi Taw 15013, Myanmar

**Keywords:** winter wheat, ryegrass, alfalfa, forage quality, nutritional values, harvesting stages, mixed-cropping

## Abstract

Forage has a significant association with animal nutrition because it is an essential part of milk and meat production in the livestock industry. Thus, for the production of high-quality forage, cereal–legume mixed cropping is an efficient method for meat and milk production in the livestock sector. In a two-year experiment between 2020 and 2021, the forage yield, nutritional compositions, amino acid profile, and forage quality were evaluated in the mixed cropping of winter wheat and ryegrass with alfalfa. In this study, a split-plot design with a randomized block design was employed with three sampling replicates. Cultivars were harvested at three maturity stages, namely, flowering, milk, and soft dough, depending on the wheat growth stage. The experimental results show that wheat 2 (Baomai 9)–alfalfa and ryegrass–alfalfa mixed cropping produced higher fresh biomass output than mono-cropping of wheat and ryegrass harvested at the flowering stage. Furthermore, the dry matter (DM) percentage range increased from 20.18% to 36.39%. By contrast, crude protein, crude fiber, neutral detergent fiber, and acid detergent fiber were higher at the flowering stage than at other harvesting stages with DM values of 14.28%, 34.12%, 55.06%, and 32.55%, respectively. Ryegrass–alfalfa mixing yielded higher values of mineral compositions, and T5 (Baomai 9–alfalfa) generally achieved more extraordinary amino acid compositions. The results demonstrate that wheat and ryegrass with alfalfa mixed cropping, and harvesting at the flowering period produces high-quality forage. Additionally, mixed cropping with alfalfa remarkably affected forage quality parameters, while mixed cropping of wheat cultivar 2 (Baomai 9) and alfalfa obtained the highest dry matter intake, digestible dry matter, relative feed value, total digestible nutrient, relative forage quality, and quality index values of 2.56, 68.54, 136.49, 60.50, 127.41, and 1.69, respectively. Thus, the mixed-cropping of wheat and ryegrass with alfalfa forage is recommended for its maximized quality forage production and nutritional values in livestock feedstuff.

## 1. Introduction

Currently, the demand for livestock goods is speedily intensifying with the increasing population. The livestock sector’s share of agricultural GDP grew by 33% in a short period. Animal husbandry has a crucial influence on increasing farmer revenue in China [1]. Thus, efficient and new animal production alternatives are required to satisfy rising food requirements. Innovative production approaches should be developed for the steady and long-standing expansion of animal production. Forage mainly influences milk and meat production in the livestock industry. If fodder does not accomplish the demand in terms of both superiority and abundance, it may affect animal nutrition [2]. Thus, the development of high-quality fodder and the search to enhance production practices such as cropping systems should be focused on. The integration of ideal species combination is a well-planned mixed cropping system for fodder production, and it provides numerous benefits, such as promoting yield and quality of forage and increasing land-use efficiency.

Legumes are widely employed in intercropping and mixed cropping systems, such as intercropping with grass to expand harvest and are proposed as not only for first-class feed sources but for yield advantages [3,4,5]. Among the 100 combinations in integrated cropping, 70% of cultivars are leguminous species utilized in China’s intercropping method [6]. Alfalfa is the superlative crop among legume crops for sustaining soil fertility because of its nitrogen-fixing capacities. It complements high protein, vitamins, and minerals, making alfalfa a good legume fodder. Alfalfa is the king of fodder crops [7]. Advanced dry matter output and forage superiority can be obtained by intercropping grass with alfalfa as a superlative instance. Wheat (*Triticum aestivum*) is China’s third most substantial and extensively harvested crop next to rice and maize [8,9]. Wheat may be a convenient supply of high-quality fodder throughout late winter and early spring, especially when the harvesting and superiority of other forage feed foundations are usually inadequate. Winter wheat is used to produce forage hay because of its higher crude protein (CP) and dry matter digestibility (DMI) than alfalfa [10]. Annual ryegrass (*Lolium multiflorum* L.) is a commonly exploited fodder grass, which is expansively grown up throughout Europe, America, and Asia, and has been developed as the foremost feed source for herbivorous animals, especially in the winter season because of its outstanding forage distinction [11]. Furthermore, ryegrass is widely used in crop rotation and intercropping systems with legumes species, because advanced crude protein content and nutrient concentration will be possible in forage.

Mixed cropping may provide enriched agronomic yields and forage production, control weed issues, diminish soil erosion, decrease insect and disease invasion, and restore soil fertility in the case of legumes [12,13,14]. When energy- and protein-rich species are mixed in forage production and given to the animals, the output of animals may be increased, as shown by many studies. In addition, many researchers evaluated the advantages of the mixed cropping of cereals or grasses and protein-rich leguminous species [15,16]. Although limited studies have focused on the forage of winter wheat, most cereal–legume studies for forage production are related to maize and small grain cereals. However, in some literature, the positive effects could be achieved in wheat by intercropping with leguminous crops for forage production. For example, forage quality is improved when clover is intercropped with wheat, thus increasing neutral detergent fiber (NDF) and acid detergent fiber (ADF) levels [17]. Additionally, more excellent dry matter content, higher crude protein percent, and advanced water-soluble carbohydrates were obtained when wheat was intercropped with beans rather than sole wheat [3]. Furthermore, Italian Ryegrass and forage pea mixed cropping contributed to higher crude protein and dry matter content, and cultivation stability was improved [18]. Additionally, the most excellent mixed cropping system with alfalfa and ryegrass enhanced total seasonal DM production, reduced weeds, increased NDF concentration, improved digestibility, raised N concentration, and improved water-soluble carbohydrate (WSC) concentration [19]. Considering that alfalfa and ryegrass are perennial and annual crops, a smaller ratio of ryegrass in mix cropping can be used for the stability of forage production of alfalfa in subsequent years. Huggaard et al., reported that intercropping with alfalfa can improve the biomass of both forage cultivars, including alfalfa, because of its characteristics [20]. Thus, alfalfa an essential leguminous crop, can effectively support another crop in the mixed cropping system. Favorable dry matter production and feed quality have been reported in combining timothy and alfalfa cropping [21]. Moreover, Tuantuan et al., demonstrated that intercropping of ryegrass and alfalfa could promote plant biomass yield and reduce heavy metal contamination in contaminated soil [22].

Harvesting time is one of the furthermost significant aspects that determines the excellence of forage feed. Timely harvesting is critical for ensuring the most excellent content and diminishing the risk of mould infection in the field [23]. Diverse harvesting times may disturb the superiority of fodder, particularly the nutritional contents of forage [24]. Crops that are harvested in the vegetative stage for forage have a minor production and fiber content, but the highest digestibility at the dry matter and nutrient compositions level are available when harvested at reproductive stage [25]. Considering these findings, the yield and nutrient composition of forage produced from mixed cropping of winter wheat–alfalfa and ryegrass–alfalfa and the effect of harvest time on yield and nutritional quality of forage were studied.

## 2. Materials and Methods

### 2.1. Experimental Site

**Figure 1:**
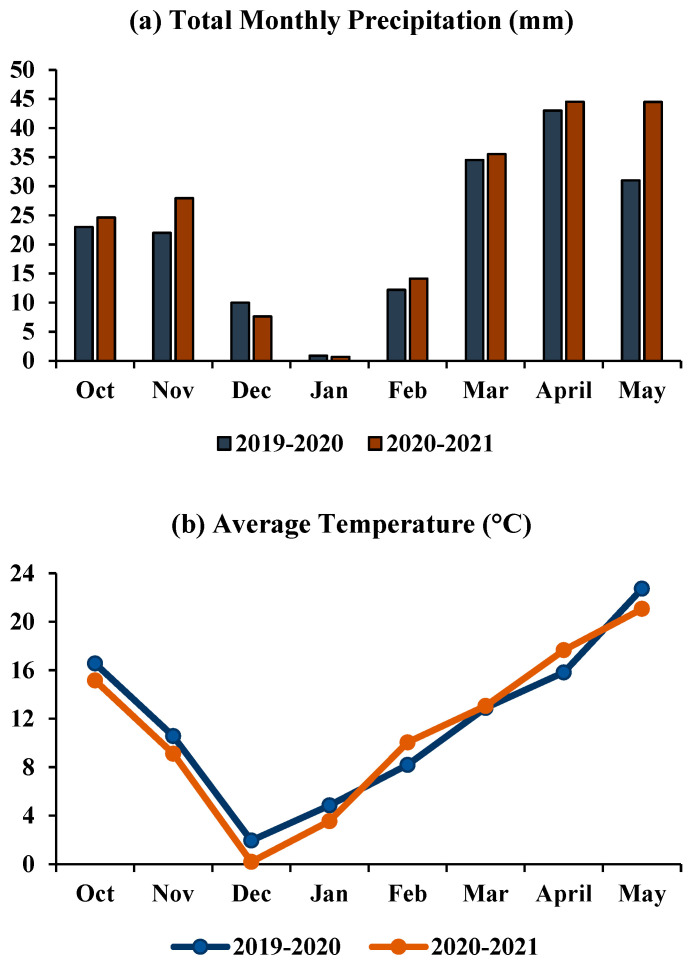
Meteorological data recorded during the 2019–2020 and 2020–2021 growing seasons; (**a**) total monthly precipitation, (**b**) monthly average temperature.

The study was conducted at the Doukou Wheat and Maize Demonstration Research Station, Northwest A&F University, Shaanxi Province, China, during the wheat cropping seasons in 2019–2020 and 2020–2021. The station is located at 108° 52′ E, 34° 37′ N and 435 m above sea level. The Campbell scientific system was used to accumulate meteorological data (weather data). During the cropping season, the weather was cooler, and less rain was recorded compared with previous years. The annual average temperature and annual precipitation were 11.46 °C and 597.06 mm, respectively (Figure 1).

The soil is a kind of Earth-cumuli-Orthic Anthrosol [26]. The seedbeds were prepared by ploughing and harrowing. Before sowing, soil samples at a depth of 0–20 cm were randomly collected using a soil auger from five randomly chosen locations within the experimental sites and evaluated for chemical characteristics after air drying, grinding, and screening [27]. The average results revealed for both cropping seasons total nitrogen content of 1.29 g kg^−1^, phosphorus level of 18. 83 mg kg^−1^, available potassium level of 232.07 mg kg^−1^, organic matter content of 18.02 mg kg^−1^, and pH of 7.9, indicating medium fertility.

### 2.2. Experimental Treatments, Design and Forage Cultivation

**Figure 2:**
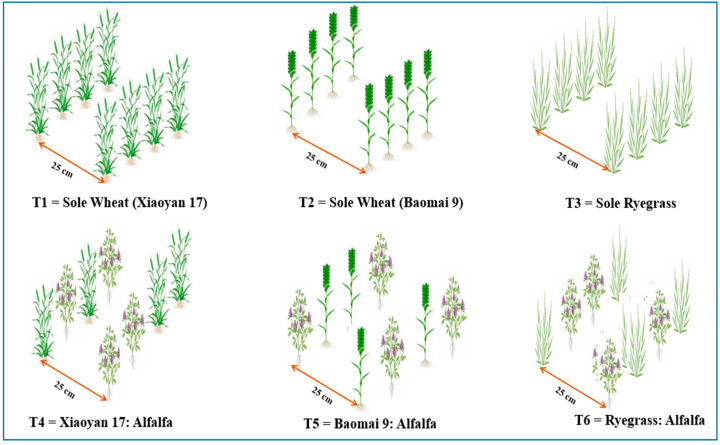
Schematic diagram of the sole cropping of winter wheat (Xiaoyan 17 and Baomai 9), sole cropping of ryegrass, winter wheat cultivar 1 (Xiaoyan 17)–alfalfa mixed cropping, winter wheat cultivar 2 (Baomai 9)–alfalfa mixed cropping, and ryegrass–alfalfa mixed cropping.

Two commercial hybrid winter wheat varieties (Xiaoyan 17 and Baomai 9, a high tillering capacity and a frost resistance variety, respectively), alfalfa (a frost-resistant cultivar), and annual ryegrass were used for the study. Doukou Wheat and Maize Demonstration Research Station supplied all the test seed materials. The split-plot design in a randomized complete block design with three replicates was employed. The main plot treatments and subplots were mono-cropped and mixed-cropped forages, and harvesting time. The plot dimensions were 3 m × 7 m with 25 cm row spacing, and a drilling system was used for sowing at all trials. The alfalfa and wheat and ryegrass sowing rates were 24 and 240 kg ha^−1^, respectively. The seed ratios of mixed-cropping were set according to the local seed ratio of 10:1 for wheat mix-cropped with alfalfa and ryegrass–alfalfa mixed cropping. All the seeds were seeded synchronously during the growing seasons on 5 October 2020 and 21 October 2021. Six different planting patterns, namely, sole cropping wheat Xiaoyan 17 cultivar (T1), sole cropping wheat Baomai 9 cultivar (T2), sole cropping ryegrass (T3), wheat Xiaoyan 17 mixed cropping with alfalfa (T4), wheat Baomai 9 mixed-cropping with alfalfa (T5), and ryegrass–alfalfa mixed cropping (T6), were employed. The experimental cropping pattern is depicted in Figure 2.

The summer maize was the previous crop before the winter wheat cropping season of 2020 and 2021. Firstly, 0.2–0.3 m plant depth ploughing was carried out for land preparation, and seeds were sown by manual hand drilling method following the furrow line. For mixed cropping, cereal and legume seeds were thoroughly mixed and drilled in the trials. Three bottles of 40% Chlorpyrifos (300 mL) were provided by mixing with 45 kg ha^−1^ wheat bran to all trials to prevent soil pests before sowing. Based on the soil test results, the same amounts of the recommended fertilizer rate were for all plots for basal fertilization by using the manual fertilizer spreader (AM-001100, Acme Agro-Tech Co., Ltd., Hubei, China) where the fertilizer contained 288 kg ha^−1^ urea (CH₄N₂O) and 288 kg ha^−1^ di-ammonium phosphate ((NH_4_)_2_HPO_4_). The irrigation was thoroughly applied twice at the tillering and stem elongation stages. Herbicides and insecticides were not used. Weeding was continuously performed by hand on all plots uniformly. No pest and disease infections were observed throughout the cropping seasons.

### 2.3. Sample Collection and Sample Preparation

Each cultivar was harvested according to the growth stage of winter wheat (cereal). The harvesting times and stage of development of each cultivar are shown in Table 1. The forage (1 m^2^) was harvested to the ground level with manual shears to collect the biomass samples in each plot. Firstly, the above-ground plant part from each harvested plot was weighted to record the fresh biomass yield. Then, the sample was dried to constant weight. Afterwards, the fresh biomass and the dry matter yield for 1 ha area were calculated by converting t ha^−1^. Approximately 500 g of fresh samples was collected from the harvested forage of each trial, and the selected sample was chopped into a length of 3–4 cm by using a power chaff cutter (JB 400, Surat, India) and dried in an oven (101-3AB Air Dry Oven, Tianjin Tester Instrument Co., Ltd., Tianjin, China) at 85 °C for 48 h to determine the dry matter percent. Then, the dried samples were powdered with a grinder (FW, interior-1 Taiwan, Tianjin Xinbode Instrument Co., Ltd., Tianjin, China) and passed through a 1 mm sieve (BL–earth-soil sieve, Shanghai Baolan Experimental Instruments Manufacturing Co., Ltd., Shanghai, China) to check the proximate composition and forage quality. Nitrogen concentration was determined using a Kjeldahl Analyzer (Hanon Shandong Scientific Instruments Co., Ltd., Jinan, China). Afterwards, crude protein percent (CP%) was calculated by multiplying by 6.25 to nitrogen concentration [28]. Crude protein percent was changed to t ha^−1^ for crude protein yield.

### 2.4. Proximate and Mineral Compositions and Amino Acid Profiles Determination

The water-soluble carbohydrate (WSC) was determined using the anthrone reaction rate essay [29]. Ether extract (EE) was evaluated via the method of the Soxhlet extraction procedure by using a Soxhlet Extractor (Hanon Shandong Scientific Instruments Co., Ltd., Jinan, China) [30]. The ground samples were burned at 550 °C for 3 h in a 12 L Stainless Steel Ceramic Muffle Furnace (Faithful Instruments Co., Ltd., Chanzhou, China) to evaluate the ash content [31]. Crude fiber (CF), NDF, and ADF were evaluated using the ANKOM 200 fiber analyzer (ANKOM Technology, Macedon, NY, USA). Mineral concentrations (Ca, Na, K, P and Mg) were determined using the AOAC method [31]. Essential amino acid (AA) profiles for forage (arginine, histidine, isoleucine, leucine, lysine, methionine, phenylalanine, threonine, and valine) were analyzed using the AOAC official method [31]. In brief, individual AA concentrations were analyzed after hydrolysis in 6 N HCl/2% phenol at 110 °C for 22 h in an amino acid analyzer (Model L-8900, Hitachi, Chyoudaku, Japan).

### 2.5. Laboratory Forage Quality Analysis

DMI was calculated using the formula DMI (% of BW) = 120/(NDF,% of DM), and digestible dry matter (DDM) was calculated using the formula (% of DM) = 88.9 − 0.779 × ADF (% of DM) [16,32]. Total digestible nutrient was calculated as TDN = 111.8 − (0.95 × % CP) − (0.36 × % ADF) − (0.7 × % NDF); relative feed value (RFV) was computed as RFV = [(120/NDF) × (88.9 − 0.779 × ADF)]/1.29; relative forage quality (RFQ) was calculated as FFQ = (DMI,% of BW) × (TDN,% of DM)/1.23; and quality index was calculated as QI = 0.0125 × RFQ + 0.097 [16,32].

### 2.6. Statistical Analysis

Triplicates were used for all trial tests. The agronomic yield, proximate, mineral composition and AA profile, forage quality, scatter matrix, and heatmap correlation data were analyzed using one-way ANOVA on SPSS software (version 22, IBM Co., Chicago, IL, USA). Duncan’s test (*p*-value 0.05) was performed to compare the treatment means. Graphs were generated using Excel 2016.

## 3. Results

### 3.1. Fresh Biomass Yield

**Figure 3:**
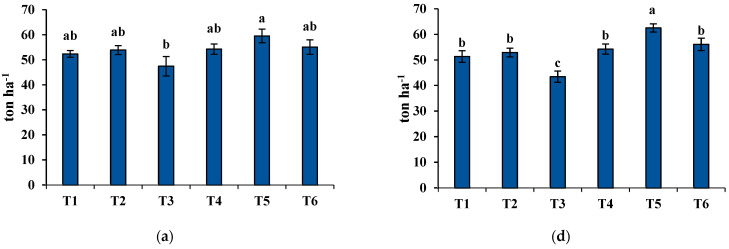

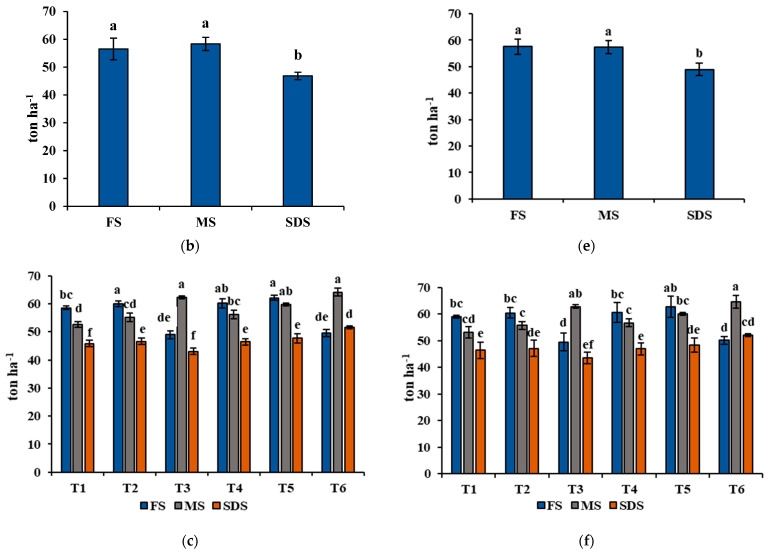
Agronomic parameter, fresh biomass yield (FBY) collected during both growing seasons. Explanations: (**a**) treatment (year 2020); (**b**) harvesting times (year 2020); (**c**) interaction between treatment and harvesting times (year 2020); (**d**) treatment (year 2021); (**e**) harvesting time (year 2021); (**f**) interaction between treatment and harvesting times (year 2021). The treatment is specified as follows: T1 = wheat cultivar 1 (Xiaoyan 17) sole cropping; T2 = wheat cultivar 2 (Baomai 9) sole cropping; T3 = ryegrass sole cropping; T4 = wheat cultivar 1 (Xiaoyan 17) with alfalfa mixed cropping; T5 = wheat cultivar 2 (Baomai 9) with alfalfa mixed cropping; T6 = ryegrass with alfalfa mixed cropping, and the harvesting time is indicated as FS = harvested at flowering stage of cereal (wheat); MS = harvested at milk stage of cereal (wheat); SDS = harvested at soft dough stage of cereal (wheat). Different letters show significant differences at *p* < 0.05. Bars point to the standard deviation.

One of the main agronomic factors, average fresh biomass yield (FBY), which was recorded throughout the 2020 and 2021 growing seasons, is described in Figure 3. The highest FBY was found in T5, which was mixed cropping of wheat 2 (Baomai 9) and alfalfa, while the lowest was found in T3 (sole cropping of ryegrass). In addition, mixed cropping with alfalfa remarkably affected the fresh biomass production among the group. Based on the comparison between T2 and T5, and T3 and T6, the biomass yield was slightly higher in mixed cropping with alfalfa than the sole cropping. However, there was no significant difference between T1 and T4. Considering harvest time, the highest FBY was recorded at the flowering and milk stages, and the lowest was observed at the soft dough stage. For the interaction between treatment and harvest time, mixed cropping with alfalfa was suitable in fresh forage biomass production, and harvesting at the flowering and milk stages resulted in higher FBY for all treatments.

### 3.2. Dry Matter Yield

**Figure 4:**
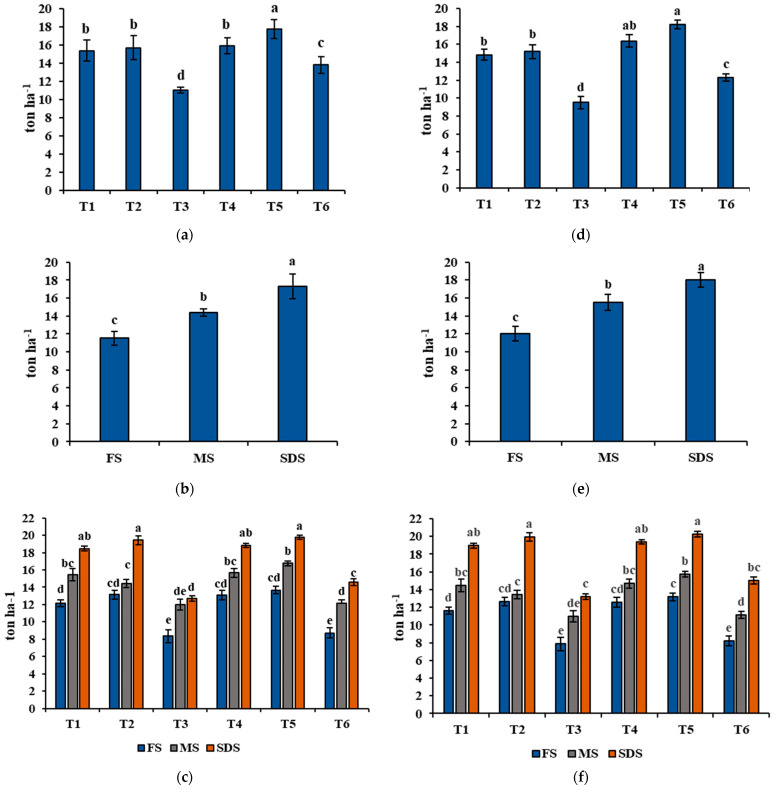
Agronomic parameter, dry matter yield (DMY) recorded during the 2020 and 2021 growing seasons. Explanations: (**a**) treatment (year 2020); (**b**) harvesting times (year 2020); (**c**) interaction between treatment and harvesting times (year 2020); (**d**) treatment (year 2021); (**e**) harvesting time (year 2021); (**f**) interaction between treatment and harvesting times (year 2021). The treatments are indicated as follows: T1 = wheat cultivar 1 (Xiaoyan 17) sole cropping; T2 = wheat cultivar 2 (Baomai 9) sole cropping; T3 = ryegrass sole cropping; T4 = wheat cultivar 1 (Xiaoyan 17) with alfalfa mixed cropping; T5 = wheat cultivar 2 (Baomai 9) with alfalfa mixed cropping; T6 = ryegrass with alfalfa mixed cropping, and the cutting time is indicated as FS = harvested at flowering stage of cereal (wheat); MS = harvested at milk stage of cereal (wheat); SDS = harvested at soft dough stage of cereal (wheat). Different letters express significant differences at *p* < 0.05. Standard deviation specified by bars.

The DMY of wheat and ryegrass sole cropping and mixed cropping with alfalfa throughout the 2020 and 2021 growing seasons are shown in Figure 4. The results showed that mixed cropping with alfalfa acquired a higher DMY than the sole cropping for all treatments. DMY was significantly affected between the treatments. The highest DMY was established in T5, whereas the lowest value was obtained at T3. Significant differences in DMY were found between the harvesting stage, harvesting at the soft dough stage resulted in the highest value, and harvesting at the flowering stage resulted in the lowest value. Considering the interaction between the treatments and harvesting stages, the highest DMY was recorded in the mixed cropping, and the soft dough stage obtained a higher DMY than in other harvesting stages.

### 3.3. Crude Protein Yield

**Figure 5:**
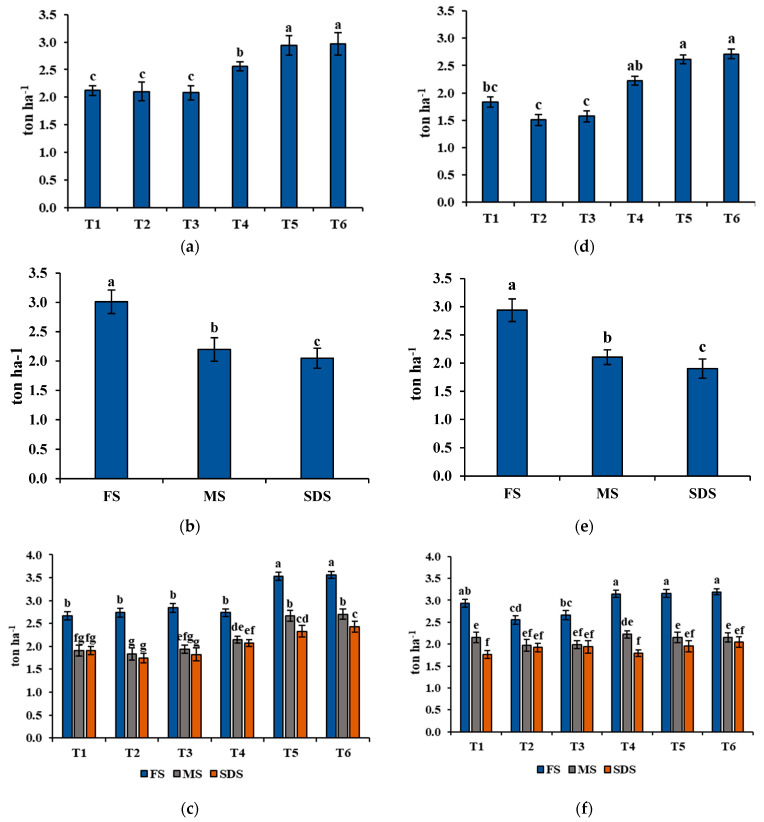
Crude protein yield (CPY) was recorded during the 2020 and 2021 growing seasons. Explanations: (**a**) treatment (year 2020); (**b**) harvesting times (year 2020); (**c**) interaction between treatment and harvesting times (year 2020); (**d**) treatment (year 2021); (**e**) harvesting time (year 2021); (**f**) interaction between treatment and harvesting times (year 2021). The treatments are indicated as follows: T1 = wheat cultivar 1 (Xiaoyan 17) sole cropping; T2 = wheat cultivar 2 (Baomai 9) sole cropping; T3 = ryegrass sole cropping; T4 = wheat cultivar 1 (Xiaoyan 17) with alfalfa mixed cropping; T5 = wheat cultivar 2 (Baomai 9) with alfalfa mixed cropping; T6 = ryegrass with alfalfa mixed cropping, and the cutting time is indicated as FS = harvested at flowering stage of cereal (wheat); MS = harvested at milk stage of cereal (wheat); SDS = harvested at soft dough stage of cereal (wheat). Different letters express significant differences at *p* < 0.05. Standard deviation specified by bars.

The crude protein yield (CPY) collected from the 2020 and 2021 growing seasons is expressed in Figure 5. The result evaluated that mixed cropping with alfalfa remarkably affected the treatments in which T6 (ryegrass–alfalfa mixed cropping) and T5 (Baomai 9–alfalfa mixing) expressed the highest CPY in both growing seasons. Moreover, the 2020 growing season resulted in a higher CPY value than the 2021 growing season. Low CPY was observed in the sole cropping of wheat and ryegrass. Additionally, harvesting at the flowering stage yielded the outstanding value of CPY. Based on the treatment and harvesting stage interaction, the highest CPY yield was established in T5 (Baomai 9–alfalfa mixing) and T6 (ryegrass–alfalfa mixed cropping) harvested at the flowering stage in both years.

### 3.4. Scatterplot Matrix Analysis of the Forage of Wheat, Ryegrass Mono-Cropping and Wheat, Ryegrass Mixed Cropping with Alfalfa

**Figure 6:**
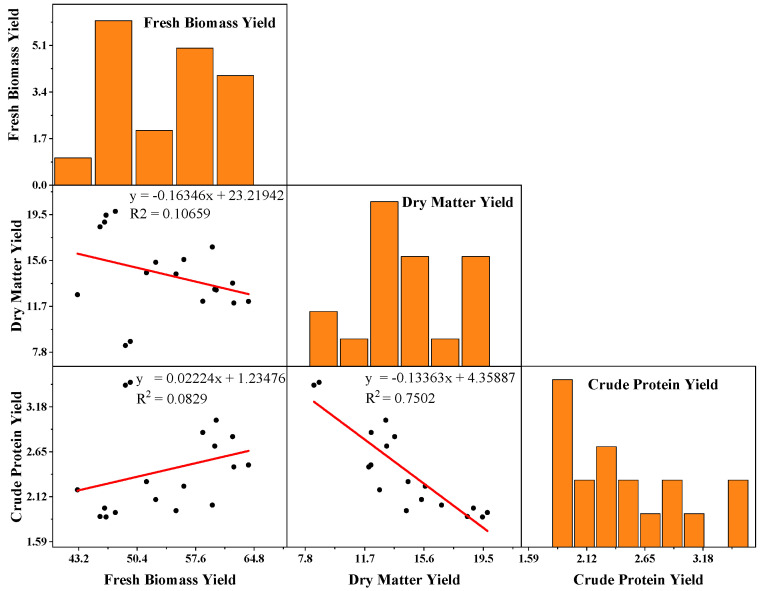
Scatterplot matrix including equation and regression values for pairwise correlation analyses between fresh biomass yield (FBY), dry matter yield (DMY), and crude protein yield (CPY) of forage conducted by wheat and ryegrass mono-cropping and wheat and ryegrass mixed cropping with alfalfa. Diagonal boxes showed histograms for each variable. The lower triangular matrix shows the relationship between a pair of variables.

Figure 6 describes the scatterplot matrix analysis of fodder of FBY, DMY, and CPY to observe and realize the relationship between different variables of FBY, DMY, and CPY of wheat and ryegrass sole cropping and mixed cropping with alfalfa. FBY was positively correlated with CPY and it was negatively associated with DMY when DMY showed a strong negative correlation with CPY.

### 3.5. Proximate and Mineral Composition Analysis

Table 2 demonstrates the principal parameters of forage proximate composition, namely, DM%, CP%, ash, CF%, EE%, WSC%, NDF%, and ADF%, which were determined during the 2020 and 2021 growing seasons. The mixed cropping of winter wheat (Baomai 9) with alfalfa resulted in the highest DM% (30.77%) and CF% (32.30%), while ryegrass mono-cropping resulted in the lowest DM% (20.81%) and CF% (27.32%). The maximum CP%, WSC%, EE%, ash, NDF%, and ADF% (12.86%, 13.67%, 2.75%, 10.47%, 57.06%, and 34.27%) were observed in ryegrass–alfalfa mixtures, whereas the lowest CP%, WSC%, EE%, NDF%, and ADF% (10.20%, 10.23%, 2.43%, 47.56%, and 26.13%) was observed in wheat 1 (Xiaoyan 17) sole cropping, and the concentration of ash had no significance differences between the wheat cultivars sole cropping and mixed cropping of wheat cultivars with alfalfa. Thus cereal–legume (alfalfa) mixed cropping significantly affected the proportions of proximate analysis based on the comparison between T1 and T4, T2 and T5, and T3 and T6. Based on the harvesting stages and influence of year, remarkable concentrations of CP, WSC, EE, CF, ash, NDF, and ADF were obtained when harvesting at the flowering stage of wheat. The highest DM% was obtained after harvesting at the soft dough stage. The 2020 growing season obtained high concentrations of all nutritional parameters.

The mineral contents (Ca, Na, K, P, and Mg) of wheat and ryegrass sole cropping and alfalfa mixed cropping are shown in Table 3. The highest mineral compositions (Ca, Na, K, P, and Mg) were recorded in ryegrass and alfalfa mixed cropping, while wheat 1 (Xiaoyan 17) had the lowest Ca and Na concentrations. Therefore, the ryegrass–alfalfa combination had a noticeable influence on mineral compositions. Furthermore, wheat–alfalfa mixed cropping enhanced the mineral contents (Ca, Na, K, and P). Baomai 9 with alfalfa mixing yielded greater mineral concentrations than the wheat varieties. Harvesting at the flowering stage led to high mineral compositions (Ca, K, P, and Mg) compared with the wheat growth stage. However, harvesting at the milk stage resulted in higher Na concentration than in other harvest stages. The least mineral compositions were obtained at the soft dough stage. No significant influence was observed over the growing seasons.

### 3.6. Amino Acid Profiles

Table 4 highlights the essential AA contents (arginine, histidine, isoleucine, leucine, lysine, methionine, phenylalanine, threonine, and valine) of wheat and ryegrass sole cropping and mixed cropping with alfalfa harvested at various stages of growth. A substantial mixed cropping effect was observed in AA compositions. The ryegrass–alfalfa mixed cropping produced high levels of isoleucine, lysine, and phenylalanine, while wheat 2 (Baomai 9)–alfalfa mixed cropping produced high levels of histidine, leucine, threonine, methionine, and valine. The most negligible value for most AA contents was observed in the sole-cropped wheat 1 (Xiaoyan 17). Ryegrass–alfalfa mixing did not affect arginine levels compared with ryegrass sole cropping. High AA concentrations were observed in Baomai 9–alfalfa mixing and ryegrass–alfalfa mixed cropping. In terms of harvesting time, substantial amounts of arginine, histidine, isoleucine, leucine, lysine, phenylalanine, threonine, and valine were obtained when harvesting at the flowering stage. Methionine had the highest perceived value when harvesting at the milk stage of wheat. Additionally, higher AA concentrations were mainly observed in the 2020 growing season compared with 2021.

### 3.7. Forage Quality Analysis

Table 5 shows the forage quality parameters (DMI, DDM, RFV, TDN, RFQ, and QI) harvested at different maturity stages. Wheat and ryegrass and alfalfa significantly influenced mixed cropping, as indicated by the higher DMI, DDM, RFV, TDN, RFQ, and QI obtained in T5 (Baomai 9 and alfalfa mixed cropping) and T6 (ryegrass and alfalfa mixed cropping) compared with the sole cropping of cereals. Mixed cropping of wheat 2 (Baomai 9) and alfalfa obtained the maximum values of DMI, DDM, RFV, TDN, RFQ, and QI (2.56, 68.54, 136.49, 60.50, 127.41, and 1.69, respectively), while the lowest values of 2.11, 62.21, 101.59, 47.30, 81.08, and 1.11, respectively, were obtained from wheat 1 (Xiaoyan 17) sole cropping. In addition, ryegrass mono-cropping showed higher forage quality values between the two wheat varieties, and wheat 2 (Baomai 9) yielded higher forage quality parameters than wheat 1 (Xiaoyan 17). Harvesting at the flowering stage yielded the highest DMI, DDM, RFV, TDN, RFQ, and QI values. This study showed that the 2020 growing season achieved significant DDM, RFV, TDN, RFQ, and QI values. The quality parameters value of ryegrass was lower than those of wheat crop.

### 3.8. Correlation Analysis of Proximate Compositions and Forage Quality Parameters

**Figure 7:**
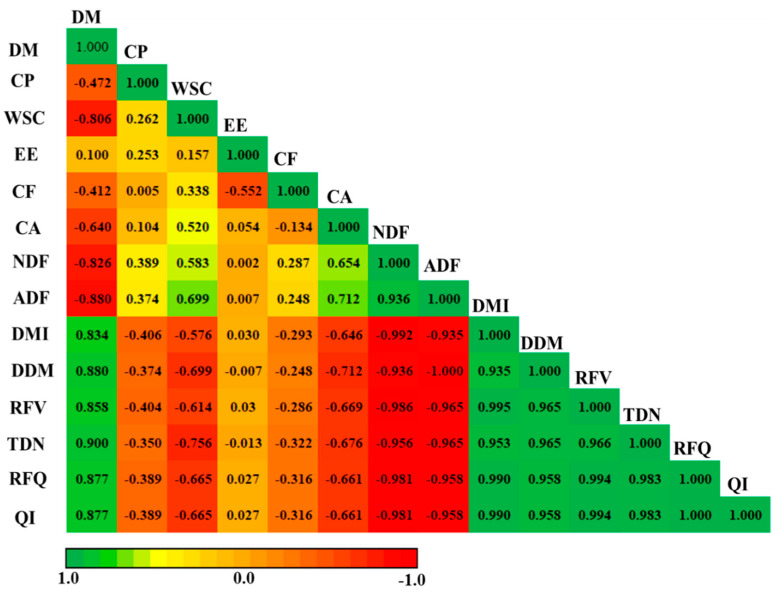
Pearson’s correlation coefficient between nutritional composition and quality parameters. DM = dry matter, CP = crude protein, WSC = water-soluble carbohydrates, EE = ether extract, CA = crude ash, CF = crude fiber, NDF = neutral detergent fiber, ADF = acid detergent fiber, DMI = dry matter intake, DDM = digestible dry matter, RFV = relative feed value, TDN = total digestible nutrient, RFQ = relative forage quality, QI = quality index. Significant correlation (*p* < 0.05) is represented by the color; the deeper the color of the field is, the more significant the correlation (*p* < 0.01). The green color signifies a positive correlation, and the red color means a negative correlation.

Correlation analysis was conducted to understand the relationship between the nutritional composition and forage quality parameters of wheat and ryegrass sole cropping and wheat and ryegrass mixed cropping with alfalfa, and the results are shown in Figure 7. A significant negative correlation was observed between DM and WSC, CP, CA, CF, NDF, and ADF. ADF was positively correlated with NDF, whereas NDF was negatively correlated with DMI. Furthermore, a negative association was observed in TDN with ADF and NDF. RFV was also negatively correlated with NDF and ADF, whereas RFQ was positively associated with DMI, TDN, and RFV. Similarly, QI had a highly positive interaction with RFQ.

## 4. Discussion

### 4.1. Assessment of FBY, Dry Forage Yield, and CPY

The present study showed that the FBY and DMY of winter wheat and annual ryegrass were higher in mixed cropping with alfalfa than in sole cropping at all harvesting stages. Generally, alfalfa is a good feed grass, with seven times the protein content and more than twice the digestible energy of wheat grain, as well as high amounts of vitamins, minerals, and trace elements and the ability to enhance reproductive and growth hormone activity. Alfalfa can also fix nitrogen. Mixed cropping with alfalfa has resulted in various consequences. In maize–alfalfa intercropping, Grabber [33] and Berti et al. [34] discovered that alfalfa has a favorable effect on maize biomass yield. According to Amaraei et al. [21], intercropping alfalfa with grass effectively increases fodder DMY in grazing areas. Additionally, intercropping alfalfa with wheat boosts wheat output significantly by enhancing weed control [35]. Moreover, Be’langer et al. [19] evaluated that timothy’s DMY was similarly higher in alfalfa–timothy intercropping than solitary cropping.

On the monocultures of alfalfa, orchard grass, and tall fescue in British Columbia, grass–alfalfa mixtures yielded more outstanding results than sole cropping [36]. According to Jalil [37], the lowest forage output was obtained at a seed ratio of 0:100 between alfalfa and ryegrass, while the best forage production was harvested at seed ratios of 80:20 and 60:40 between alfalfa and ryegrass. Based on the present study, alfalfa provided the grass crops nutrients, thus increasing the biomass and dry matter yields. However, some studies have demonstrated the consequences of the intercropping system’s competition. Intercropping allows crops to effectively utilize light, water, and nutrients and alter crop biomass. This outcome is based on intercropped component changes in the competitive variance capability for development aspects. Sustainable agricultural production systems can be created through the effective use of available growth resources.

Furthermore, in an intercropping system, the optimum plant density of each intercropped component is also a critical issue to avoid competition amongst crops, especially regarding nutrients, water, and shading effects. In the present study, the plant density of alfalfa per unit area was a 10:1 seed proportion of alfalfa to grass crops. The overall yield of a mixture is remarkably affected by the density of mixed crops per unit area, which is the factor that is most affected by competition [38]. Thus, in the present study, the lower seed rate of alfalfa in mixing enhanced forage yield and nutrient compositions of forage. In addition, forage quantity and quality were affected by the environmental effects. According to Berti and Samarappuli [39], reduced grain and biomass yields were observed under alfalfa intercropping with corn because of the intercropped alfalfa’s competition for water, which is a critical component for maize growth. For intercropped elements, alfalfa is an outstanding choice for intercropping with winter wheat and annual ryegrass to increase biomass and dry forage output, according to our study. In addition, CP yield remarkably differed among wheat–alfalfa, ryegrass–alfalfa, and mono-cropping of wheat and ryegrass. Soe Htet et al. [40] concluded that cultivation techniques are positively related to the CP yield of forage. Wheat cultivar 2, Baomai 9–alfalfa mixing, and ryegrass–alfalfa mixed cropping showed a higher CP yield than the wheat sole and ryegrass sole amongst treatments. Moreover, the 2020 growing season showed a higher CPY value than the 2021 growing season because of the preferable weather situation in the 2020 growing season for plant growth.

The biomass yield and forage DMY were also affected by harvesting stages. In the present research, FBY declined as the harvest time approached, supporting previous research on numerous herbage crops that linked this tendency to leaf senescence and stored carbohydrate remobilization, resulting in a fall in plant biomass after flowering [41]. Furthermore, the outcomes of the harvesting periods revealed that fresh biomass output is more significant at the milk stage than the flowering stage, because ryegrass development is slower than wheat and is much lower at the soft dough stage. DMY increased from the flowering stage to dough phases, as predicted and supported by the study of Francia et al. [42] on triticale grown in Northern Italy. Increased DMY was observed for various forage crops approaching harvest time, including perennial ryegrass, Italian ryegrass, tall fescue, cocksfoot, timothy, and red clover [43]. Harvesting forage at soft dough for cereal crops is the best time to satisfy the desired dry forage yield. Additionally, a high CPY value was observed when harvesting at the flowering stage in the current study. Similarly, forage wheat tended to have higher CP yield at the early harvesting stage because of higher plant density and plant height in the mixed cropping of wheat and ryegrass, according to Xu et al. [44]. They recommended that multiple harvesting or grazing several times could provide more benefits for improving CP yield in the ryegrass cultivar. These findings are associated with the plant development cycle and photosynthetic storage in the grain.

### 4.2. Evaluation of Nutritional Compositions (Proximate and Mineral) and Amino Acid Profiles

The DM range for cereal sole cropping is 20.8–30.05, while cereal legume mixed cropping has a DM range of 23.16–30.77. Additionally, intercropping with alfalfa resulted in advanced nutritious compositions of CP, WSC, EE, and ash concentrations more than mono-cropping. Zhang et al. [45] discovered that total biomass and harvest material nutritional balance could be improved when alfalfa is incorporated with a standard cereal rotation system. Furthermore, they concluded that the combinations of legume and grasses cropping could enhance the available nutrient compositions in terms of DM and CP. Additionally, the ratio of CP increased in legume species when intercropping trials were undertaken in various crops, according to the discussion of numerous investigations. According to Lithourgidis et al. [46], intercropping cereals with legumes has several compensations over monocultures, including higher DM, enhanced land-use efficiency and crop yield stability, superior consumption of light and nutrients, and improved soil conservation. The mixture of alfalfa–timothy and alfalfa sole cropping was studied by Be’langer et al. [19]. They showed that combining timothy and alfalfa enhanced DM output, reduced weed invasion, and produced a more favorable WSC:CP ratio. Furthermore, high WSC concentration was recorded in alfalfa and timothy mixing. However, a lower CP concentration of the alfalfa–timothy mixture was recorded when comparing the mono-cropping of timothy. The high content of WSC resulted in the efficient utilization of CP by feeding to cows and can promote milk production. Soe Htet et al. [16] also implied the crop fodder quality characteristics of CP and ash content in the study of climbing bean and soybean mixed cropping compared with the sole cropping of climbing bean. Conversely to our study, the lower essential nutritive compositions, especially CP, was recorded in the study of alfalfa–tall fescue mixture [36]. Generally, grass mixing with legumes improved the nutritive quality compared with grasses solitary cropping; grass–legume mixtures had a remarkably higher total N and mineral and fiber content than grasses, although legumes usually have a lower fiber concentration in the percent of DM compared with grasses [47]. In the present study, the nutrient compositions of grass/alfalfa mixtures were similar to those of the other previous grass–legumes intercropping, although the proportion of alfalfa was lower in mixing.

Furthermore, grass–alfalfa mixed cropping affected the fiber concentrations, which is an essential forage parameter. In the current study, CF, NDF, and ADF concentration decreased in wheat and ryegrass sole cropping compared with wheat–alfalfa and ryegrass–alfalfa mixed cropping. Furthermore, the higher contents of NDF and ADF resulted in ryegrass–alfalfa mixtures. In the same way, Contreras-Gova et al. [17] evaluated that wheat–clover intercropping increased fodder quality in terms of NDF and ADF concentration compared with wheat sole cropping. This may be because of the higher ratio of grass in the mixture; thus, grasses have higher fiber content compared with legume species. Conversely to our study, Sleugh et al. [48] reported a 30% reduction in NDF levels in Kura clover–wheat grass intercropping compared to sole cropping. Moreover, Kunelius et al. [49] found that mixtures containing red clover or alfalfa obtained a lower NDF level amongst grass–legumes mixed cropping. In addition, a relatively low CF concentration was observed in the sole cropping of ryegrass. Therefore, the high energy content could be observed in the ryegrass cultivar compared with other cultivars. Kunelius et al. [49] found a low fiber concentration of a perennial ryegrass mixture. Additionally, in the current study, CP was positively correlated with imperative parameters, namely, NDF and ADF. By contrast, CP was adversely correlated with CF and NDF, according to Soe Htet et al. [40] and Chaudhary et al. [50]. Although DM showed adverse relationships with nutritive parameters, including CP, NDF, and ADF, it had positive interactions with forage quality parameters. Be’langer et al. [19] suggested an upgraded relationship between DM and nutritive concentrations with no adversative effects on forage quality by mixing timothy and alfalfa.

In the present study, ryegrass–alfalfa mixed cropping attained high mineral concentrations, and harvesting at the flowering stage resulted in high mineral compositions (Ca, K, P, and Mg), except for Na. Pirhofer-Walzl et al. [51] found high mineral compositions in terms of K, Mg, Ca, Mn, and Fe in the mixture of grass–legume herb compared with the grass monoculture. In comparison with the present results, several mineral concentrations significantly increased in the mixture of herbs and grasses from the first harvesting to the third harvesting stage, while mineral compositions of legumes constantly persisted. Considering the AA compositions, substantial differences were observed in AA compositions, especially isoleucine and leucine, among the treatments. Moreover, the AA composition of alfalfa in mixed cropping slightly influenced the AA contents in the current study. Limited studies have focused on the effects of the cropping system on AA compositions; a few reports of AA compositions in some species are available [52]. Furthermore, most AA compositions obtained a higher value at the flowering stage. This result may be appropriate because of the higher vegetative proportion of plant parts in the early harvesting stage. Similarly, Liu and Mahmood [53] reported slightly higher essential AA compositions of alfalfa leaves and orchard grass compared with those of the whole plant.

In addition, the nutritious compositions of small grain cereal forages are heavily influenced by the time of harvest. In the current research, the DM had a higher value of advancing maturity, but the CP had a lower value. Borreani et al. [54] investigated the production and quality of semi-leafless grain peas (*Pisum sativum* L.) and found that DM increased as maturity progressed, whereas CP decreased considerably. Other papers that studied the influence of harvest time on alfalfa revealed a reduction in CP as maturity progressed [55]. In the intercropping of winter wheat and bean (*Vica faba* L.), they revealed that the similar result in the decreasing of CP advancing maturity [38]. Jacobs and Ward [56] discovered that bi-cropping forage peas with winter cereal forage crops did not reduce DM yields at various harvest periods and did not increase nutritional characteristics consistently and significantly. Furthermore, the harvesting stages of any crop are significantly related to NDF levels by promoting the increased cell wall components, particularly cellulose, hemicelluloses, and lignin [57]. Fiber concentrations (whether ADF or NDF) were improved until the flowering time but began to fall (in some cases, considerably) after substantial seed development, and TDN usually increases when harvesting at the milk stage to the soft dough stage [58]. In the present study, a high TDN value was observed at the flowering stage. According to this study’s nutritive parameters, the ideal time to harvest winter wheat is until the grain milk stage, which is consistent with data on maize and cowpea (*Vigna unguiculate* L.) [58]. Moreover, our results also support the authors’ findings that wheat silage should be harvested at the flowering stage to obtain the maximum nutritional values, particularly CP, NDF, and ADF. The chemical composition of any forage/grass and the animals’ ability to digest it determines the intake [59].

### 4.3. Forage Quality Analysis

Our findings revealed that cereal legume mixed cropping produced excellent forage quality parameters, such as RFV, RFQ, and QI. According to the potential digestible dry matter intake, the relative feed value index (RFV) rates cool-season legumes, grasses, and combinations. It allows for the proper feed distribution to the appropriate livestock class with a specified degree of expected performance. The quality index (QI) is a comprehensive measure of fodder quality. RFQ is a better form of RFV. When forage is fed as the sole source of energy and protein, it is an estimate of voluntary intake of available energy. It also includes fiber digestibility and quantity measurements. DMI as a percentage of BW, as in RFV, is the intake component, while TDN (percent of DM), as in QI, is the available energy component. DMI is the percentage of body weight estimate of how much feed an animal will consume. Differences in CP and cell wall (ADF and NDF) contents could explain the variability in DDM and TDN readings [16,60]. A comparable outcome of our study was found in an experiment on the nutritional value and palatability of several range types of grass. They concluded that grasses’ QI and RFQ values ranged from 1.41 to 1.8 and from 105.08 to 138.36 percent, respectively [60]. Moore and Daniel concluded that the value of QI is less than 1.0 and expressed that low-quality forage and weight loss would be expected [32]. Thus, the QI range of 1.11–1.69 in the current study shows the high quality of forage. Additionally, when hairy vetch (*vicia villosa*) and Columbus grass (*sorghum almum*) were mixed, the RFV, RFQ, and QI values for solitary Columbus grass were more significant than mixed cropping with hairy vetch. RFV values below 100 are considered lower than the essential beginning point, which is RFV 100, according to the RFV scale. Dairy cows that produce a lot of milk require a diet with an RFV of at least 130 [60].

## 5. Conclusions

With this study, we have established that mono-cropping and mixed cropping of winter wheat with ryegrass and alfalfa at different harvesting stages affects the fresh fodder yield, DMY, nutritional compositions, and quality of each crop species in the mixtures. Thus, it ensures the best source of nutritionally rich forage to support livestock feeding. Harvesting at the flowering stage is recommended for all treatments to obtain outstanding chemical compositions. Furthermore, Baomai 9 was preferable as a forage crop to attain a higher forage yield and dry forage yield compared with Xiaoyan 17 wheat cultivar. Among the mixed-cropping treatments, Baomai 9 and alfalfa mixed cropping showed the most significant values of forage quality indexes, namely, RFV, TDN, RFQ, and QI. Sole cropping between two wheat varieties, Xiaoyan 17 Baomai 9, and ryegrass were compared, and ryegrass was recommended as a high-quality forage crop because of its high contents of nutrient compositions in terms of CP, NDF, and ADF and high forage quality values. Further studies should investigate the different management practices for grasses (wheat and ryegrass) and alfalfa mixed cropping to be promoted in the production of high-quality silage and animal performance for feeding in the livestock industry and then evaluate different harvesting stages of forage for crop production.

## Figures and Tables

**Table 1:** Harvesting time and development stages of wheat, ryegrass, and alfalfa.

Forage Species	Harvesting Stage	Harvested Date
Wheat 1 (Xiaoyan 17)	Flowering stage	20 March
Wheat 2 (Baomai 9)	Flowering stage	20 March
Ryegrass	Booting stage	20 March
Alfalfa	Late vegetative stage	20 March
Wheat 1 (Xiaoyan 17)	Milk stage	3 April
Wheat 2 (Baomai 9)	Milk stage	3 April
Ryegrass	Flowering stage	3 April
Alfalfa	Early bud stage	3 April
Wheat 1 (Xiaoyan 17)	Soft dough stage	18 April
Wheat 2 (Baomai 9)	Soft dough stage	18 April
Ryegrass	Early heading stage	18 April
Alfalfa	Late bud stage	18 April

**Table 2:** Proximate compositions of forage were conducted by wheat and ryegrass sole-cropped and mixed with alfalfa harvested at different maturity stages (DM%).

Treatment	Parameters (%)							
	DM	CP	WSC	EE	CF	Ash	NDF	ADF
T1	29.36 b	10.20 d	10.23 e	2.43 e	31.92 b	7.72 c	47.56 f	26.13 e
T2	30.05 b	10.67 c	11.20 d	2.48 d	31.79 c	7.62 c	49.35 e	28.01 d
T3	20.81 d	12.62 a	13.24 c	2.55 c	27.32 e	10.31 b	54.66 b	32.71 b
T4	30.32 b	10.56 c	13.52 b	2.48 d	32.20 a	7.55 c	50.31 d	29.45 c
T5	30.77 a	11.29 b	13.40 b	2.61 b	32.30 a	7.79 c	53.58 c	29.47 c
T6	23.16 c	12.86 a	13.67 a	2.75 a	30.17 d	10.47 a	57.06 a	34.27 a
SEM	0.27	0.11	0.71	0.01	0.18	0.16	0.44	0.30
LOS	*	**	**	**	**	*	**	**
Harvest Stage								
Flowering Stage	20.18 c	14.28 a	15.60 a	2.61 a	34.12 a	9.10 a	55.06 a	32.55 a
Milk Stage	25.66 b	10.33 b	12.64 b	2.50 c	30.35 b	8.41 b	52.86 b	30.35 b
Soft Dough Stage	36.39 a	9.49 c	9.41 c	2.53 b	28.38 c	8.22 b	48.35 c	27.10 c
SEM	0.19	0.08	0.05	0.01	0.12	0.11	0.31	0.21
LOS	**	**	**	**	**	*	**	**
Year								
2020	28.49 a	11.62 a	12.63 a	3.08 a	31.24 a	8.64 a	53.22 a	31.25 a
2021	26.33 b	11.12 b	12.47 b	2.02 b	30.66 b	8.52 b	50.95 a	28.76 b
SEM	0.16	0.50	0.04	0.29	0.53	0.33	1.38	0.91
LOS	**	**	ns	*	ns	ns	ns	*

DM = dry matter; CP = crude protein; ash; CF = crude fiber; EE = ether extract; WSC = water-soluble carbohydrates; NDF = neutral detergent fiber; ADF = acid detergent fiber. SEM = standard error; ns = non-significant; * = significant differences at the 0.05 level and ** = significant differences at the 0.01 level. Different letters express the values that significance different at 0.05 probability level. T1 = wheat cultivar 1 (Xiaoyan 17) sole cropping; T2 = wheat cultivar 2 (Baomai 9) sole cropping; T3 = ryegrass sole cropping; T4 = wheat cultivar 1 (Xiaoyan 17) with alfalfa mixed cropping; T5 = wheat cultivar 2 (Baomai 9) with alfalfa mixed cropping; T6 = ryegrass with alfalfa mixed cropping. Flowering stage = harvested at flowering stage of cereal (wheat); milk stage = harvested at milk stage of cereal (wheat); soft dough stage = harvested at soft dough stage of cereal (wheat).

**Table 3:** Mineral composition of fresh forage of wheat mono-cropping, ryegrass sole cropping, and wheat and ryegrass mixed with alfalfa harvested at different maturity stages (DM%).

Treatment	Parameters (%)				
	Ca	Na	K	P	Mg
T1	0.20 c	0.03 c	2.03 c	0.27 b	0.08 d
T2	0.21 c	0.04 c	2.01 c	0.26 b	0.07 d
T3	0.31 b	0.14 b	2.02 c	0.27 b	0.30 b
T4	0.21 c	0.04 c	2.06 c	0.27 b	0.10 c
T5	0.31 b	0.14 b	2.13 b	0.27 b	0.30 b
T6	0.40 a	0.20 a	2.46 a	0.32 a	0.50 a
SEM	0.001	0.008	0.05	0.01	0.001
LOS	*	*	*	*	*
Harvest Stage					
Flowering Stage	0.27 a	0.08 a	2.48 a	0.33 a	0.22 a
Milk Stage	0.26 a	0.09 a	2.06 b	0.27 b	0.19 b
Soft Dough Stage	0.23 b	0.07 a	1.82 c	0.23 c	0.15 c
SEM	0.006	0.001	0.04	0.01	0.001
LOS	*	ns	**	**	**
Year					
2020	0.25 a	0.06 a	2.10 a	0.28 a	0.18 a
2021	0.26 a	0.09 a	2.14 a	0.28 a	0.20 a
SEM	0.02	0.03	0.05	0.01	0.05
LOS	ns	ns	ns	ns	ns

Ca = calcium; Na = sodium; K = potassium; P = phosphorus; Mg = magnesium. SEM = standard error; ns = non-significant; * = significant differences at the 0.05 level and ** = significant differences at the 0.01 level. Different letters express the values that significance different at 0.05 probability level. T1 = wheat cultivar 1 (Xiaoyan 17) sole cropping; T2 = wheat cultivar 2 (Baomai 9) sole cropping; T3 = ryegrass sole cropping; T4 = wheat cultivar 1 (Xiaoyan 17) with alfalfa mixed cropping; T5 = wheat cultivar 2 (Baomai 9) with alfalfa mixed cropping; T6 = ryegrass with alfalfa mixed cropping. Flowering stage = harvested at flowering stage of cereal (wheat); milk stage = harvested at milk stage of cereal (wheat); soft dough stage = harvested at soft dough stage of cereal (wheat) and fodder types as fresh forage and hay.

**Table 4:** Amino acid profile of fresh forage and hay of wheat and ryegrass sole-cropped and mixed with alfalfa harvested at different maturity stages (DM%).

Treatment	Amino Acid Profile (%)								
	Arg	His	Ile	Leu	Lys	Met	Phe	The	Val
T1	7.1 d	2.5 c	4.0 e	7.1 e	5.6 d	1.3 c	5.6 c	5.0 c	4.9 d
T2	7.3 c	2.6 b	4.0 e	7.1 e	5.7 d	1.0 d	6.2 b	5.4 b	6.5 b
T3	7.7 a	2.6 b	5.1 b	8.7 c	6.0 c	1.9 b	6.2 b	5.1 c	6.3 c
T4	7.2 c	2.5 c	4.2 d	8.5 d	6.4 b	1.5 c	6.2 b	5.0 c	6.3 c
T5	7.5 b	2.8 a	4.5 c	9.8 a	6.4 b	2.7 a	7.0 a	6.0 a	7.0 a
T6	7.1 d	2.7 a	5.5 a	9.0 b	6.8 a	1.8 b	7.1 a	5.6 b	6.8 a
SEM	0.02	0.02	0.02	0.02	0.03	0.03	0.01	0.01	0.02
LOS	*	*	**	**	*	*	*	*	*
Harvest Time									
Flowering Stage	7.5 a	2.9 a	4.9 a	11.6 a	6.3 a	1.8 b	6.9 a	5.9 a	6.5 a
Milk Stage	7.1 b	2.4 b	4.6 b	9.2 b	6.2 a	2.1 a	6.2 b	4.9 c	6.2 b
Soft Dough Stage	7.5 a	2.5 b	4.2 c	9.2 b	5.5 b	1.2 c	6.0 c	5.3 b	6.1 b
SEM	0.01	0.01	0.01	0.01	0.02	0.03	0.01	0.02	0.02
LOS	*	*	**	*	*	**	**	**	*
Year									
2020	7.1 b	2.6 a	4.7 a	10.3 a	6.6 a	1.7 a	6.3 a	5.4 a	6.6 a
2021	7.6 a	2.6 a	4.4 b	9.4 b	5.6 b	1.7 a	6.4 a	5.3 a	5.9 b
SEM	0.01	0.02	0.04	0.03	0.03	0.02	0.02	0.01	0.02
LOS	**	ns	*	*	**	ns	ns	ns	*

Arg = arginine; His = histidine; Ile = isoleucine; Leu = leucine; Lys = lysine; Met = methionine; Phe = phenylalanine; Thr = threonine; Val = valine. SEM = standard error; ns = non-significant; * = significant differences at the 0.05 level and ** = significant differences at the 0.01 level. Different letters express the values that are significantly different at the 0.05 probability level. T1 = wheat cultivar 1 (Xiaoyan 17) sole cropping; T2 = wheat cultivar 2 (Baomai 9) sole cropping; T3 = ryegrass sole cropping; T4 = wheat cultivar 1 (Xiaoyan 17) with alfalfa mixed cropping; T5 = wheat cultivar 2 (Baomai 9) with alfalfa mixed cropping; T6 = ryegrass with alfalfa mixed cropping. Flowering stage = harvested at flowering stage of cereal (wheat); milk stage = harvested at milk stage of cereal (wheat); soft dough stage = harvested at soft dough stage of cereal (wheat) and fodder types as fresh forage and hay.

**Table 5:** Forage quality of forage cultivated by wheat and ryegrass sole-cropped and mixed with alfalfa harvested at different maturity stages (DM%).

Treatment	Quality Indexes (Values)					
	DMI	DDM	RFV	TDN	RFQ	QI
T1	2.11 f	62.21 e	101.59 f	47.30 f	81.08 f	1.11 f
T2	2.20 e	63.42 d	108.13 e	50.98 e	91.24 e	1.24 e
T3	2.39 c	65.96 c	122.65 c	55.92 c	109.42 c	1.46 c
T4	2.27 d	65.94 c	116.83 d	54.00 d	101.34 d	1.36 d
T5	2.56 a	68.54 a	136.49 a	60.50 a	127.41 a	1.69 a
T6	2.46 b	67.08 b	128.71 b	57.08 b	115.52 b	1.54 b
SEM	0.03	0.24	2.05	0.48	2.57	0.03
LOS	**	**	**	**	**	**
Harvest Stage						
Flowering Stage	2.52 a	67.79 a	134.91 a	59.59 a	124.02 a	1.65 a
Milk Stage	2.28 b	65.25 b	115.93 b	54.38 b	101.39 b	1.36 b
Soft Dough Stage	2.19 c	63.54 c	108.33 c	48.92 c	87.59 c	1.19 c
SEM	0.21	0.17	1.45	0.34	1.82	0.02
LOS	**	**	**	**	**	**
Year						
2020	2.38 a	66.50 a	123.33 a	55.64 a	109.04 a	1.46 a
2021	2.28 a	64.56 b	114.81 b	52.95 b	99.62 b	1.34 b
SEM	0.06	0.71	4.48	1.16	4.98	0.06
LOS	ns	*	*	*	*	*

DMI = dry matter intake; DDM = digestible dry matter; RFV = relative feed value; TDN = total digestible nutrient; RFQ = relative forage quality; QI = quality index. SEM = standard error; ns = non-significant; * = significant differences at the 0.05 level and ** = significant differences at the 0.01 level. Different letters express the values that are significantly different at the 0.05 probability level. T1 = wheat cultivar 1 (Xiaoyan 17) sole cropping; T2 = wheat cultivar 2 (Baomai 9) sole cropping; T3 = ryegrass sole cropping; T4 = wheat cultivar 1 (Xiaoyan 17) with alfalfa mixed cropping; T5 = wheat cultivar 2 (Baomai 9) with alfalfa mixed cropping; T6 = ryegrass with alfalfa mixed cropping. Flowering stage = harvested at flowering stage of cereal (wheat); milk stage = harvested at milk stage of cereal (wheat); soft dough stage = harvested at soft dough stage of cereal (wheat).

## Data Availability

All obtained data are enclosed with this manuscript.
